# TiO_2_/graphene/CuSbS_2_ mixed-dimensional array with high-performance photoelectrochemical properties†

**DOI:** 10.1039/c9ra07237c

**Published:** 2019-10-21

**Authors:** Qianyuan Chen, Zhongchi Wang, Keqiang Chen, Qiang Fu, Yueli Liu, Yupeng Zhang, Delong Li, Chunxu Pan

**Affiliations:** School of Physics and Technology, MOE Key Laboratory of Artificial Micro- and Nano-structures, Wuhan University Wuhan 430072 China cxpan@whu.edu.cn; Institute of Microscale Optoelectronics, College of Electronic Science and Technology, Shenzhen Key Laboratory of Flexible Memory Materials and Devices, Shenzhen University Shenzhen 518060 China ypzhang@szu.edu.cn ldlong19890809@163.com; State Key Laboratory of Silicate Materials for Architectures, School of Materials Science and Engineering, Wuhan University of Technology Wuhan 430070 China; Center for Electron Microscopy, Wuhan University Wuhan 430072 China

## Abstract

The growing demands for reproducible and clean sources of power has prompted the exploitation of novel materials for solar-energy conversion; in any case, the improvement of their conversion efficiency remains a big challenge. We report a mixed-dimensional heterostructure to synchronously enhance charge separation and light-absorption of the photoanodes *via* the introduction of two-dimensional reduced graphene oxide and zero-dimensional CuSbS_2_ quantum dots on one-dimensional TiO_2_ arrays. The experimental results show that the graphene sheets with a low Fermi level and a superior electron mobility accept photo-excited electrons from TiO_2_ and enable fast electron transportation; while the CuSbS_2_ quantum dots promote the visible light-absorption of the photoanode. The synergistic effects in this mixed-dimensional (1D–2D–0D) heterostructure photoanode induce a markedly raised photoconversion efficiency of 1.2% at 0.3 V and a photocurrent density of 5.5 mA cm^−2^ at 0.4 V. Furthermore, the photocurrent density of the mixed-dimensional heterostructure exceeds previously reported TiO_2_-based photoanodes in neutral media. The improved photoelectrochemical properties are attributed to the synergistic-effect-induced highly organized, mixed-dimensional architectures. It is expected that the mixed-dimensional heterostructure photoanode will be a potential candidate for applications in environmental remediation and energy fields.

## Abstract 2

The growing demands for reproducible and clean sources of power has prompted the exploitation of novel materials for solar-energy conversion; in any case, the improvement of their conversion efficiency remains a big challenge.

## Graphical abstract

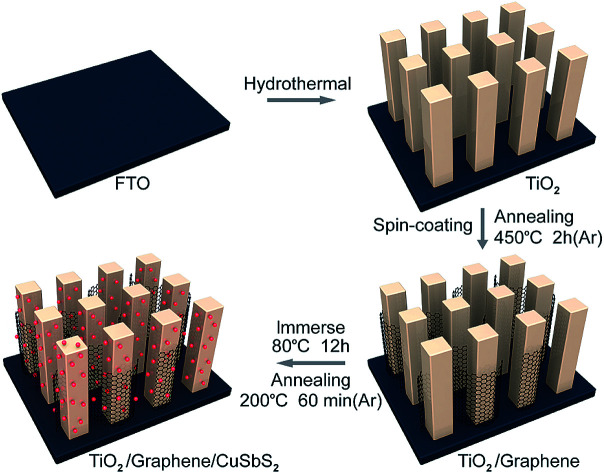

## 1 Introduction

Solar energy is regarded as a feasible alternative to fossil fuels because of its promise for addressing environmental remediation and energy conversion. Therefore, the ability to efficiently harness and utilize solar energy is of great importance. Since its use for water photolysis by Fujishima and Honda in 1972, TiO_2_ has garnered considerable attention in solar cells and photoelectrochemical devices.^1–6^ However, TiO_2_ exhibits several drawbacks, such as the fast recombination of photo-excited carriers and a low-degree of quantum efficiency (monochromatic incident photon-to-electron conversion efficiency). Meanwhile, TiO_2_ can only convert less than 5% of the full solar spectrum and this limits the use of solar energy as a primary source of energy because TiO_2_ is a wide band-gap semiconductor (3.2 eV).^7–12^

Great efforts have been made including the geometry and band structure engineering to solve these problems. For instance, one-dimensional (1D) TiO_2_ including TiO_2_-tubes, TiO_2_-rods, TiO_2_-fibers, TiO_2_-belts, and TiO_2_-wires recently have attracted wide interest for photoelectrical applications because of their large surface area for light-absorption, as well as an axial transport of photo-generated carriers with fast transport property.^13–18^ In addition, forming a Schottky or p–n junctions to realize band structure engineering is another effective approach, which aims to widen the visible light-absorption and hinder from the recombination of electrons and holes to improve the photon-to-energy conversion efficiency.^19–21^ For example, coupling 1D TiO_2_ with narrow bandgap zero-dimensional (0D) quantum dots could enhance the photoconductivity by photogating effect. Furthermore, the dangling-bond surfaces of 2D graphene materials offer flexibility in integrating different dimensioned materials into mixed-dimensional heterostructures for next-generation electronic/optoelectronic devices.^22^ The introduction of two-dimensional (2D) materials such as graphene into the 1D TiO_2_, will promote the carrier transfer efficiency. The synergistic effect fully exploits the extraordinary electron transport characteristics of 2D graphene and further makes large contribution to the highly promoted photoelectrochemical performance of the mixed-dimensional heterostructures device. It is thus of strategic interest to synthesize the 0D quantum dots/1D TiO_2_ nanowires/2D graphene (mixed-dimensional) heterostructures with appropriate composition and well-designed structure.^23–27^ The interface in this mixed-dimensional heterostructure is less constrained due to the absence of the need for lattice matching, resulting in effective interaction.

Recently, copper antimony sulfide (Cu–Sb–S) quantum dots have gained significant attention in solar cells and photoelectrochemical devices.^28^ As a p-type semiconductor with a high light-absorption coefficient (over 105 cm^−1^ at visible wavelength), copper antimony sulfide quantum dots are made up from environmentally-friendly and earth-abundant elements, the major phase of Cu–Sb–S has a bulk band-gap in the range of 1.0–1.8 eV; this type of non-toxic quantum dots have a high potential for photosensitization of 1D TiO_2_. Specifically, graphene is widely used in photocatalysis for its unique atomic-thick 2D structure and super physicochemical properties. When combined with 0D the quantum dots/1D TiO_2_ heterostructure, owe to 2D graphene higher work function which induces photogenerated electron transfer from the conduction band of the semiconductor to graphene. In other word, 2D graphene acts as an electron shuttle.^29^ To enhance the surface reaction kinetics, introduction of RGO into the 0D quantum dots/1D TiO_2_ to create mixed-dimensional (0D–1D–2D) heterostructures which provide new reaction pathways to cut down kinetic barriers.

In this work, negative charged graphene oxide (GO) was self-assembled on rutile TiO_2_ arrays; then, the composite was annealed at 450 °C in an N_2_ atmosphere for 2 h to improve the adhesion between graphene and TiO_2_.^10^ Finally, the 1D TiO_2_/2D graphene/0D CuSbS_2_ quantum dots prepared by wet chemical method, followed by annealing at 200 °C for 60 min in argon (Ar) to obtain ternary 1D TiO_2_/2D graphene/0D CuSbS_2_ nanocomposites. To increase the interfacial contact between the 1D TiO_2_ arrays, 2D graphene and the 0D CuSbS_2_ quantum dots, the graphene sheets were fragmented between the quantum dot/TiO_2_ heterostructure by tuning the concentration of the spin-coated GO.^25^ As a consequence, the contact area and interaction between graphene and the other two phases was markedly increased. The experimental results show that the ternary composites exhibited excellent photoelectrochemical properties under solar light irradiation; the peak current reached 6 mA cm^−2^ and the photo-energy conversion efficiency was 1.2%. Furthermore, the graphene sheets not only reduced the open potential of photocurrent but also improved the stability of the CuSbS_2_ quantum dots under prolonged irradiation. In a word, it is expected that mixed-dimensional heterostructure photoanode will be a promising candidate for applications in environmental remediation and energy fields.

## 2 Experimental section

### 2.1 Materials

The natural graphite powder, 325 mesh, 99.95% was bought from Alfa Aesar. The fluorine-doped tin oxide (FTO) was bought from Zhuhai Kaivo Optoelectronic Technology Co., Ltd. (Zhuhai, China). The copper(i) iodide (CuI, 98%), antimony(iii) chloride (SbCl_3_, 99%), sodium chloride (NaCl, 99.9%), phosphorus pentoxide (P_2_O_5_, 99.9%), potassium thiosulfate (K_2_S_2_O_4_, 99.9%), potassium permanganate (KMnO_4_, 99.9%), titanium(iv) butoxide (Ti(OBu)_4_, 99%), *N*,*N*′-diphenylthiourea (98%), diphenyl ether (99%), oleylamine (OLA, 80–90%), hexane (C_6_H_14_, 99%), absolute ethanol (C_2_H_6_O, 99%), propan-2-ol (C_3_H_8_O, 99%), acetone (C_3_H_6_O, 99%), hydrochloric acid (HCl, 20 wt%), sulfuric acid (H_2_SO_4_, 98%), hydrogen peroxide (H_2_O_2_, 30%), were purchased from Shanghai Chemical Reagents Company without any further treatment. Deionized water was used for all the experiments.

### 2.2 Preparation of the CuSbS_2_ quantum dots

A typical hot-injection method was used for the preparation.^28^ 1.5 mM copper(i) iodide and 1.5 mM antimony(iii) chloride were loaded into a 50 ml three-neck flask containing 30 ml oleylamine. The mixture was heated to 100 °C under an Ar atmosphere, and then a clear slight yellow solution would emerge. Afterwards, the temperature of the solution was increased to the target temperatures (150 °C) and 4.5 ml 1 M *N*,*N*′-diphenylthiourea dissolved in diphenyl ether was subsequently injected into the mixture as quickly as possible. The solution was cooled to room temperature after 5 min. The mother liquor (1 ml) was added into methanol (3 ml) and centrifuged at 8000 rpm for 3 min. Hexane (4 ml) was used to redisperse the nanocrystals, and the precipitates were separated by centrifugation at 8000 rpm for 3 min. The product was further purified by several precipitation/dispersion cycles.

### 2.3 Preparation of the TiO_2_/graphene/CuSbS_2_ composites

**Fig. 1:**
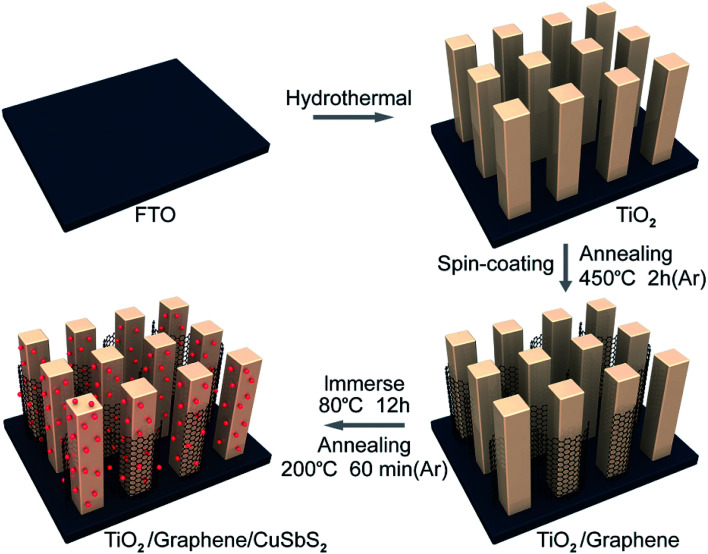
Schematic diagram of the preparation of the 0D CuSbS_2_ quantum dots/1D TiO_2_ nanowires/2D graphene (mixed-dimensional) heterostructures.

Fig. 1 illustrates the fabrication process of the ternary TiO_2_/graphene/CuSbS_2_ composites. The fluorine-doped tin oxide (FTO) substrate (10 mm × 30 mm) was ultrasonically cleaned and then the highly ordered TiO_2_ nanorod arrays were grown on the FTO substrate *via* the hydrothermal method. Briefly, 0.45 ml titanium(iv) butoxide (Ti(OBu)_4_, 99%) and 0.6 g sodium chloride (NaCl, 99.9%) were added into 30 ml diluted hydrochloric acid (HCl, 20 wt%). Then, the above precursor (30.45 ml) was transferred into a 100 ml Teflon-lined stainless steel autoclave and for hydrothermal growth at 170 °C for 8 h. The resultant samples (TiO_2_ nanowire arrays) were rinsed with deionized water and dried at 70 °C in air.

Graphene oxide (GO) was prepared *via* the modified Hummers and Offeman's method,^28^ the preparation process is given in the ESI.† The TiO_2_/GO composites were prepared by spin-coating a GO aqueous solution (1 mg ml^−1^, 350 μl) onto the TiO_2_ nanowires array substrate. Then, the composite was annealed at 450 °C in an N_2_ atmosphere for 2 h to improve the adhesion between graphene and TiO_2_.

The TiO_2_/graphene composite was immersed in the CuSbS_2_ quantum dots (3 mg ml^−1^) for 12 h (the preparation of CuSbS_2_ quantum dot was given in the ESI†), after the solvent evaporated completely, the sample was taken out and annealed at 200 °C in argon (Ar) for 60 min.

## 3 Results and discussion

### 3.1 Morphology and structure analysis

**Fig. 2:**
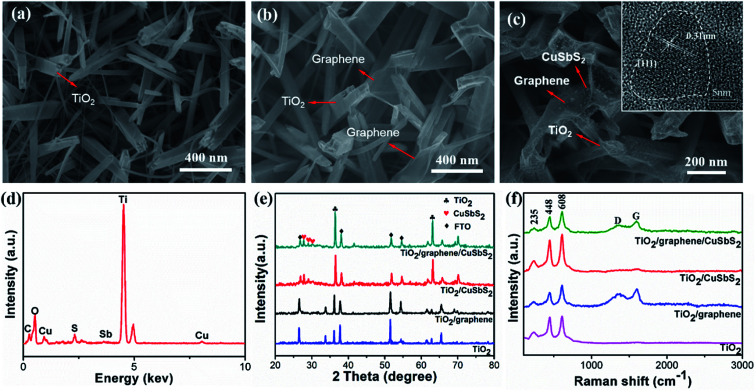
SEM morphologies of (a) 1D TiO_2_ nanowires arrays, (b) 1D TiO_2_ nanowires/2D graphene composite and (c) the 0D CuSbS_2_ quantum dots/1D TiO_2_ nanowires/2D graphene (mixed-dimensional) heterostructures (inset is TEM image of 0D CuSbS_2_ quantum dots); (d) EDX profile of the 0D CuSbS_2_ quantum dots/1D TiO_2_ nanowires/2D graphene (mixed-dimensional) heterostructures; (e) XRD patterns and (f) Raman spectra of the samples.

The scanning electron microscope (SEM) morphologies of pure 1D TiO_2_ and the 1D TiO_2_/2D graphene composite are shown in Fig. 2(a) and (b), while the 0D CuSbS_2_ quantum dots/1D TiO_2_ nanowires/2D graphene (mixed-dimensional) heterostructures are shown in Fig. 2(c). The 1D TiO_2_ arrays were prepared on the FTO substrate, as is shown in Fig. 2(a) the average diameter of 1D TiO_2_ arrays is 50–100 nm. The transparent fragmented graphene sheets were overlaid on the prepared 1D TiO_2_ arrays and made full use of the large gaps in the 1D TiO_2_ arrays. As a result, a large contact area between 1D TiO_2_ arrays and the graphene sheets was achieved (Fig. 2(b)). For the 1D TiO_2_ nanowires/0D CuSbS_2_ quantum dots composites, the CuSbS_2_ quantum dots are firmly attached to the TiO_2_ nanowires without any agglomeration (Fig. S2(a)†). Similarly, the CuSbS_2_ quantum dots are uniformly distributed on the 1D TiO_2_ nanowires/2D graphene heterostructures, as shown in Fig. 2(c) (inset is TEM image of 0D CuSbS_2_ quantum dots). The TEM image further shows the morphology of a single rod of the 0D CuSbS_2_ quantum dots/1D TiO_2_ nanowires/2D graphene (mixed-dimensional) heterostructures (Fig. S2(b)†). The EDS spectra shows uniform signals for the Ti, O, C, Cu, Sb, and S elements (Fig. 2(d)), which indicates the homogeneous distribution of the graphene and CuSbS_2_ quantum dots on the TiO_2_ nanowires.

Fig. 2(e) shows the XRD patterns of the mixed-dimensional heterostructures. The diffraction peaks of SnO_2_ at 2*θ* values of 26.578°, 37.768°, 51.755° and 65.740° (JCPDS no. 46-1088) initiated from the FTO substrate, and the diffraction peaks at 2*θ* values of 27.446°, 36.085°, 54.322° and 62.5° correspond to the (110), (101), (211) and (002) planes of rutile phase of TiO_2_ arrays, respectively (JCPDS no. 21-1276).^13^ In the TiO_2_/CuSbS_2_ and TiO_2_/graphene/CuSbS_2_ composites, the diffraction peaks at 2*θ* values of 28.47° and 32.09° correspond to the (101) and (201) planes of CuSbS_2_ quantum dots, respectively, which correspond with the HRTEM results.^30^ One should note that the rutile phase structure of the TiO_2_ nanowires shows little change after heat treatment because of its high stability. Additionally, the CuSbS_2_ quantum dots are stable in the ternary composites, and the graphene sheets exhibited no influence on the CuSbS_2_ quantum dots.^28^

The Raman spectra are shown in Fig. 2(f). The peaks at 450 cm^−1^ and 610 cm^−1^ identify the E_g_ and A_1g_ vibration modes of rutile TiO_2_, respectively.^26^ Two bands located at 1350 cm^−1^ (D band) and 1595 cm^−1^ (G band) demonstrate the presence of graphene.^31–34^ Generally speaking, the G band originates from the first order scattering of the E_2g_ mode, indicating the sp^2^ hybridized carbon atoms; the D band is assigned to the A_1g_ symmetry vibration of the K-point, revealing the sp^3^ defects in graphitic structure. Furthermore, the *I*_D_/*I*_G_ ratio is a critical indicator of the defect density or average size of the sp^2^ domains in graphene.^31^ The intensity ratio (*I*_D_/*I*_G_) of graphene oxide (GO) in the TiO_2_/GO composite was 0.97, the intensity ratio (*I*_D_/*I*_G_) decreased to 0.80 (TiO_2_/graphene) after thermal treatment, which indicated the good reduction of graphene oxide. However, the value in the TiO_2_/graphene/CuSbS_2_ composites is 0.85, which demonstrated the high surface defect density of the graphene sheets. This phenomena is ascribed to the large contact area between graphene and the CuSbS_2_ quantum dots, which induce strong interactions and led to the increased *I*_D_/*I*_G_ value.^19^

### 3.2 Optical properties

**Fig. 3:**
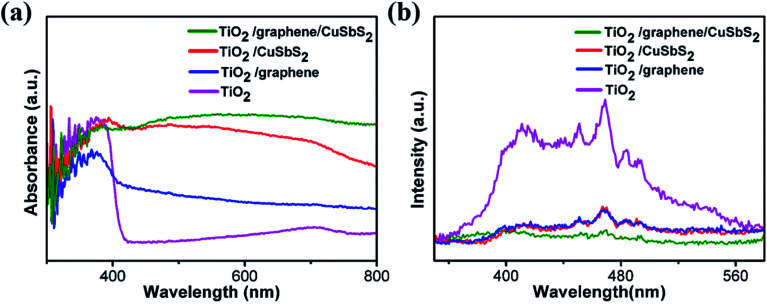
(a) UV-Vis diffuse reflectance spectra and (b) PL spectra of the samples.

The light-absorption properties of the material is a crucial factor for photoanodes. Fig. 3(a) shows the UV-Vis absorption spectra of the mixed-dimensional heterostructures. The TiO_2_ arrays exhibit strong absorption in the ultraviolet region and had an absorption edge at ∼410 nm, which corresponded to a band-gap of 3.11 eV. The 1D TiO_2_/2D graphene composite shows a higher absorption in the visible region than simplex TiO_2_ arrays, this might be ascribed to the intense visible light-absorption of graphene sheets. However, a red shift of the absorption edge was observed because the sp^2^ orbit of graphene monished the energy between the Ti (3d) and O (2p) orbits. For the 0D quantum dots/1D TiO_2_ nanowires/2D graphene (mixed-dimensional) composites, light-absorption ranged from the ultraviolet to the visible.^35,36^ Significantly, the 0D quantum dots/1D TiO_2_ nanowires/2D graphene (mixed-dimensional) composites exhibit a more intense absorption in the visible region, this might be ascribed to the large contact area between graphene and the CuSbS_2_ quantum dots, which promote the synergistic effects on light-absorption.

The PL behavior originates from the recombination of photoinduced electron–hole pairs. Fig. 3(b) illustrates the PL emission spectra of the samples; the TiO_2_ array exhibited two dominant peaks at 467 nm and 416 nm, these PL signals originate from the charge transfer and band-gap transitions of TiO_2_ due to oxygen vacancies which trap the electrons.^37,38^ The PL intensity of the 1D TiO_2_ nanowires/2D graphene and 1D TiO_2_ nanowires/0D CuSbS_2_ decline sharply because of the marriage of the graphene sheets and the fabricate the heterostructure, which observably benefit electron–hole separation.^39^ Moreover, a further fluorescence decrease was observed for the 0D quantum dots/1D TiO_2_ nanowires/2D graphene (mixed-dimensional) heterostructures photoanode, this might be attributed to the following: (1) the narrow band gap of the CuSbS_2_ quantum dots promoted the intense absorption of visible light; the potentials of the conduction band (CB) of CuSbS_2_ were more positive than TiO_2_, and the photo-generated electrons transferred to TiO_2_ because of the potential dissimilarity; (2) the higher work function of the graphene sheets promote the transfer of the photo-generated charge carriers at the interface.

### 3.3 Photoelectrochemical performance

**Fig. 4:**
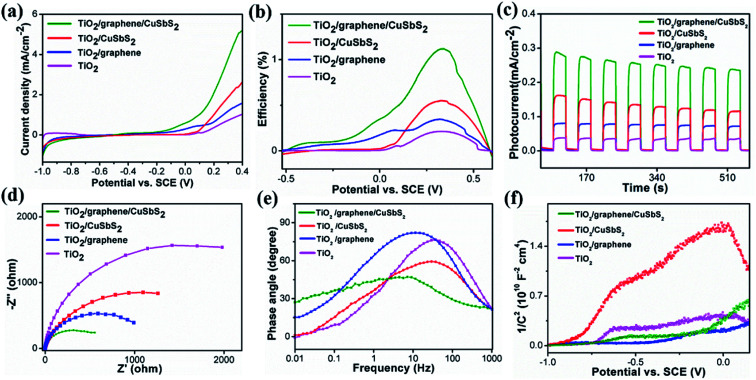
Photoelectrochemical properties of the samples: (a) current–voltage curves, (b) photoconversion efficiency curves, (c) *I*–*t* curves at a potential of 0.1 V, (d) electrochemical impedance spectra, (e) Bode phase plots, (f) Mott–Schottky plots.

Fig. 4(a) shows the results of linear sweep voltammograms (LSV). It can be seen that the TiO_2_ arrays exhibit the lowest photoresponse, with a photocurrent density of 1 mA cm^−2^ at 0.4 V and an onset potential of 0.05 V; the 0D quantum dots/1D TiO_2_ nanowires exhibited a comparable onset potential, along with an increase in the photocurrent density (2.6 mA cm^−2^ at 0.4 V) contrast to the pristine TiO_2_ arrays. This indicates that the CuSbS_2_ quantum dots were able to improve the photoresponse of the pristine TiO_2_ arrays, which could be ascribed to its high light-absorption coefficient as well as its narrow band-gap. For the 1D TiO_2_ nanowires/2D graphene, a reinforced photocurrent density (1.5 mA cm^−2^ at 0.4 V) and a cathodic shift of the onset potential relative to the pristine TiO_2_ arrays were observed. For the 0D CuSbS_2_ quantum dots/1D TiO_2_ nanowires/2D graphene (mixed-dimensional) heterostructures photoanode, the onset potential of the sample exhibit a marked negative shift. This indicated that the accumulation of photo-generated carriers at the interface is significantly reduced and could be attributed to the high charge transfer characteristics of the graphene sheets, as well as the large contact area between the graphene and the CuSbS_2_ quantum dots.^40,41^ Additionally, the photocurrent density (5.2 mA cm^−2^ at 0.4 V) increase rapidly alongside the increase of the bias voltage, which was two times higher than that of the 0D quantum dots/1D TiO_2_ nanowires (under the same bias voltage). Significantly, the photocurrent density is not saturated with increasing bias, this suggest an excellent light response and a high separation of photo-generated carriers in the 0D CuSbS_2_ quantum dots/1D TiO_2_ nanowires/2D graphene (mixed-dimensional) heterostructures, which is caused by the synergistic effects between graphene and the CuSbS_2_ quantum dots.

The photoconversion efficiency (*η*) of each photoanode was calculated based on the current voltage curve as a function of the applied voltage (Fig. 4(b)), as shown by the followed formula:^11^

1 *η* = *J* × 1.23(1.23 − *E*_RHE_)/*P*_light_

2 *E*_RHE_ = *E*_SCE_ + *E*^Θ^_SCE_ + 0.059 × pH

where *J* is the photocurrent density (mA cm^−2^), *P*_light_ is the incident light intensity (100 mw cm^−2^), *E*_RHE_ is the testing voltage relative to the reversible hydrogen electrode (RHE), *E*_SCE_ is the testing voltage relative to the SCE, and *E*^Θ^_SCE_ is the standard electrode potential of saturated calomel electrode (SCE) at 25 °C. The 0D CuSbS_2_ quantum dots/1D TiO_2_ nanowires/2D graphene (mixed-dimensional) heterostructures photoanode exhibits a highest photoconversion efficiency of 1.2% at 0.3 V, which is observably larger than that of the 1D TiO_2_ nanowires/2D graphene (0.34%), the 0D quantum dots/1D TiO_2_ nanowires (0.54%), and pristine TiO_2_ (0.2%). The PEC property of the 0D CuSbS_2_ quantum dots/1D TiO_2_ nanowires/2D graphene (mixed-dimensional) heterostructures photoanode clearly demonstrated that a synergistic effect is achieved by the integration of 2D graphene and 0D CuSbS_2_ quantum dots with 1D TiO_2_ nanowires.

Fig. 4(c) shows the transient photocurrent densities of mixed-dimensional heterostructures photoanode with bias potentials of 0.1 V under identical incident power intensities. It is found that all the mixed-dimensional heterostructures photoanode exhibit a relatively high photocurrent density rate constant compared to those of their counterparts due to the synergistic effect involving the 0D CuSbS_2_ quantum dots/1D TiO_2_ nanowires/2D graphene (mixed-dimensional) heterostructures photoanode. The photocurrent density of the TiO_2_ array is higher than that of 1D TiO_2_ nanowires/2D graphene; however, the magnitude of the photocurrent density is 20 μA. This is because of the limited use of the solar spectrum by the TiO_2_ arrays, which led to low light to energy conversion. For 1D TiO_2_ nanowires/0D CuSbS_2_ quantum dots, the photocurrent intensity of the heterojunction increase significantly to 147 μA, which indicate that the narrow band-gap CuSbS_2_ quantum dots could efficiently harness incident solar light. However, the photocurrent density is not stable, after 600 s of illumination the intensity is attenuated to 115 μA. It is important to highlight that the photocurrent density of the 0D CuSbS_2_ quantum dots/1D TiO_2_ nanowires/2D graphene (mixed-dimensional) heterostructures photoanode represent the highest value among these four photoanodes, and the photocurrent rises and falls rapidly with changes between the light-on and light-off states. Although charge relaxation was observed, this phenomenon disappeared gradually with prolonged duration. Additionally, the photocurrent density increased to 287 μA, which indicate that the graphene sheets not only effectively modulated the transport of charge carriers but also improve the stability of the CuSbS_2_ quantum dots under extended illumination. This can be attributed to the large interfacial contact area between graphene and CuSbS_2_ quantum dots. To the best of our knowledge, the photocurrent density of the 0D CuSbS_2_ quantum dots/1D TiO_2_ nanowires/2D graphene mixed-dimensional heterostructures photoanode is highest among other reported TiO_2_-based photoanodes (Table S1†) and is also the maximum of the other reported photoanodes in a neutral medium.

Electrochemical impedance spectroscopy (EIS) studies was carried out to investigate charge transport behavior in these photoanodes. Generally, a smaller semicircle diameter in the plot suggests a lower charge transfer resistance. As shown in Fig. 4(d), the novel ternary 0D CuSbS_2_ quantum dots/1D TiO_2_ nanowires/2D graphene (mixed-dimensional) heterostructures photoanode has the smallest semicircle diameter among the investigated samples. This indicate a low charge transfer resistance as well as a high electrochemical activity. The main reason for this is that graphene not only promotes the mass transfer process but also improves electron transport, which rapidly increases the carrier concentration. The Bode curves of the samples are fitted from the EIS curves to further study the effects of graphene on carrier modulation, as shown in Fig. 4(e). The mid-frequency characteristic peak of TiO_2_/graphene/CuSbS_2_ shift markedly to a lower frequency, which reveal a more rapid electron mobility process. In addition, the lifetime of electrons *τ*_eff_ can be extracted *via* the following equation:^42,43^

3 *τ*_eff_ = 1/2π*f*_min_

The electron lifetime is only determined by the minimum frequency (*f*_min_) of the mid-frequency. In actual calculations, we reverse the minimum frequency (*f*_min_) of Bode plots peak as a function of electron lifetime. The electron lifetime for the 0D CuSbS_2_ quantum dots/1D TiO_2_ nanowires/2D graphene (mixed-dimensional) heterostructures photoanode is 26.5 ms, 1D TiO_2_ nanowires/0D CuSbS_2_ quantum dots is 5.1 ms, 1D TiO_2_ nanowires/2D graphene is 15.9 ms, and for pristine TiO_2_ is 4.3 ms. The electron lifetime of the novel ternary 0D CuSbS_2_ quantum dots/1D TiO_2_ nanowires/2D graphene (mixed-dimensional) heterostructures was the highest among the samples, and it was almost 5 times higher than that of 1D TiO_2_ nanowires/0D CuSbS_2_ quantum dots. In this regard, the incorporation of graphene sheets play a vital role in modulating photo-excited carrier transport in the composites.

The semiconducting properties of the obtained samples were investigated by measuring the Mott–Schottky plots, as shown in Fig. 4(f). The positive slopes of the plots for all the samples indicate formation of an n-type TiO_2_ semiconductor. Additionally, the charge carrier density was calculated from the slope according to the following equation:^44^

4 *N*_d_ = (2/*eε*_0_*ε*)[d(1/*C*^2^)/d*V*]^−1^

where *N*_d_ is the charge carrier density, *e* is the elementary electron charge, *ε*_0_ is the permittivity in vacuum, and *ε* is the dielectric constant. The charge carrier density for the samples are 1.64 × 10^18^ cm^−3^ (TiO_2_), 5.57 × 10^18^ cm^−3^ (TiO_2_/graphene), 0.79 × 10^18^ cm^−3^ (TiO_2_/CuSbS_2_), and 5.08 × 10^18^ cm^−3^ (TiO_2/_graphene/CuSbS_2_); the results corresponded with the Bode plots, which further demonstrated the strong modulation of the charge carriers by graphene. The highly interconnected and the synergistic effects at 0D CuSbS_2_ quantum dots/1D TiO_2_ nanowires/2D graphene (mixed-dimensional) heterostructures contribute to this distinguished photoelectrochemical performance. The flat band potential is obtained by extrapolating the Mott–Schottky plot to 1/*C*^2^ = 0 to obtain the intercept. An evident positive shift could be observed after the incorporation of graphene sheets, while a negative shift occurred after the incorporation of CuSbS_2_ quantum dots. Generally, the positive shift of the flat band potential for TiO_2_ indicate a decrease in the bending of the band edges, and this could facilitate charge transfer at the electrode/electrolyte interface. The results can be ascribed to the lower Fermi level of graphene, which facilitate the transportation of charge carriers and reduce the band bending between the electrode and the electrolyte interface.

**Fig. 5:**
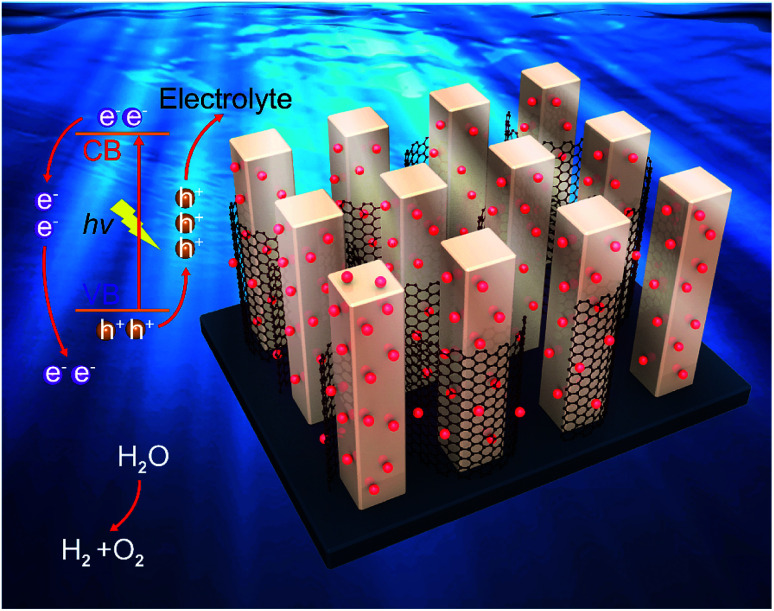
Photoelectrochemical mechanism of the 0D CuSbS_2_ quantum dots/1D TiO_2_ nanowires/2D graphene (mixed-dimensional) heterostructures.

Given the discussion above, a mechanism for the photoelectrochemical mechanism of the novel ternary 0D CuSbS_2_ quantum dots/1D TiO_2_ nanowires/2D graphene (mixed-dimensional) heterostructures is proposed and shown in Fig. 5. First, electron–hole pairs are generated in the 1D TiO_2_ nanowire arrays under illumination. Based on the structure analysis, the band energy difference between these mixed-dimensional induce both electron transfer from the 1D TiO_2_ nanowire arrays to 2D graphene and hole capture by CuSbS_2_ quantum dots. There should be a synergistic effect in the composites. This opposite transmission of electrons and holes inhibits the surface recombination of photogenerated charge carriers. The super-fast migration of electrons to the current collector was performed in 2D graphene due to its superior electron mobility, which remarkably suppresses the bulk electron–hole recombination and enhances the charge separation efficiency.^29^ We thus believe that in our mixed-dimensional heterostructures materials, the highly interconnected and the synergistic effects at different dimensional contribute to this distinguished photoelectrochemical performance. Then, a large number of photogenerated carriers (electrons and holes) are formed due to the quantum effect of the CuSbS_2_ quantum dots, the average size of CuSbS_2_ quantum dots (around 6.7 nm) is smaller than their double Debye length of the electrons, which makes the whole quantum dots become the depletion layer, and this distinguishing feature is of high absorption coefficient because of the tiny amount electron injection. Subsequently, the large specific surface area of graphene accelerates mass transfer between the electrode and electrolyte, which prompts the transfer of photogenerated holes to the electrolyte. The synergistic effect fully exploits the extraordinary electron transport characteristics of 2D graphene and further makes large contribution to the highly promoted photoelectrochemical performance of the mixed-dimensional heterostructures device.^45^ Finally, the photogenerated electrons migrate to the surface of graphene because of its lower Fermi level. Under a certain bias, the electrons collected on the graphene eventually flow to the negative pole through the TiO_2_ nanowire array under an external electric field.

## 4 Conclusions

An innovative ternary 0D CuSbS_2_ quantum dots/1D TiO_2_ nanowires/2D graphene (mixed-dimensional) heterostructures photoanode was prepared *via* a simple and economic process where graphene sheets were fragmented between 1D TiO_2_ nanowires and 0D CuSbS_2_ quantum dots. The resulting ternary nanocomposites exhibit dramatically enhanced photoelectrochemical properties, including a large photocurrent density and an extended charge carrier lifetime. The enhancement could primarily be attributed to the fragmented graphene sheets; the high work function of the graphene sheets facilitates the migration of the photo-excited charge carriers, and the synergistic effects between graphene and the 0D CuSbS_2_ quantum dots simultaneously enhances the absorption and conversion of solar energy. The demonstration of such the 0D CuSbS_2_ quantum dots/1D TiO_2_ nanowires/2D graphene mixed-dimensional heterostructures proposed here would open up a wide area of opportunities for designing high-performance photoelectrochemical device. This kind of ternary nanocomposites exhibits broad application potential in areas such as CO_2_ conversion, water splitting, gas sensors, and solar-energy conversion.

## Conflicts of interest

The authors declare no conflict of interest.

## Supplementary Material

RA-009-C9RA07237C-s001
