# Supplementary material for: TiO2/graphene/CuSbS2 mixed-dimensional array with high-performance photoelectrochemical properties
Source: RSC Adv. 2019 Oct 21;9(58):33747–54. doi: 10.1039/c9ra07237c (PMC9073703; doi:10.1039/c9ra07237c)
Supplement: RA-009-C9RA07237C-s001 [file RA-009-C9RA07237C-s001.pdf]

Electronic Supplementary Material (ESI) for RSC Advances.
This journal is © The Royal Society of Chemistry 2019

Supporting Information

Title TiO_2/graphene/$CuSbS_2$ mixed-dimensional array with high-performance photoelectrochemical properties

Qianyuan Chen[a,b], Zhongchi Wang[a], Keqiang Chen[b], Qiang Fu[d], Yueli Liu[c], Yupeng Zhang[b,*], Delong Li[b,*] and Chunxu Pan[a,d,*]

a. School of Physics and Technology, and MOE Key Laboratory of Artificial Micro- and Nano-structures, Wuhan University, Wuhan 430072, China.

b. Institute of Microscale Optoelectronics, College of Electronic Science and Technology, and Shenzhen Key Laboratory of Flexible Memory Materials and Devices, Shenzhen University, Shenzhen 518060, China.

c. State Key Laboratory of Silicate Materials for Architectures, School of Materials Science and Engineering, Wuhan University of Technology, Wuhan, 430070, China.

d. Center for Electron Microscopy, Wuhan University, Wuhan 430072, China.

* Author to whom correspondence should be addressed.

E-mail: ypzhang@szu.edu.cn (Y. Zhang), ldlong19890809@163.com(D. Li),

cxpan@whu.edu.cn (C. Pan)

Characterization methods

The morphology and microstructure of the samples were characterized via scanning electron microscope (SEM; Sirion, FEI, Netherlands), and high-resolution transmission electron microscopy (HRTEM; JEM 2010 FEF HRTEM, JEOL, Japan) equipped with an energy-dispersive X-ray spectrometer (EDS; Genesis 7000, EDAX Inc., USA). The crystalline phases were detected via an X-ray diffractometer (XRD; D8 Advance, Bruker AXS, Germany) with an angular range from 20-80°. Raman measurements were conducted on a Raman spectrometer (Raman; Jobin Yvon LabRAM HR, HORIBA, France) with a laser excitation source (λex=488 nm). The elemental compositions were analyzed via X-ray photoelectron spectroscopy (XPS; AXIS-Ultra instrument, Kratos Analytical, England) with a monochromatic Al Kα X-ray beam (225 W, 15 Ma, 15 kV). The UV-Vis diffuse reflectance spectra (DRS) of the samples were measured with the diffuse reflectance accessory of UV-Vis spectrophotometer (UV-2550; Shimadzu, Kyoto, Japan), in which $BaSO_4$ was used as a background between 200-800 scopes. The photoluminescence measurements were conducted on a Raman spectrometer (Raman; Jobin Yvon LabRAM HR, HORIBA, France) with a laser excitation wavelength at $\lambda ex = 325$ nm.

Photoelectrochemical performance

Photoelectrochemical measurements were performed on an electrochemical analyzer (CHI-660C, CH Instruments Co.) in a standard three-electrode configuration with different photoelectrodes, i.e., pristine TiO_2, TiO_2/graphene, and the TiO_2/graphene/$CuSbS_2$ composites. In addition, the Na_2SO_4 (0.1 mol L^{-1}) aqueous solution was used as an electrolyte.

The counter electrode and the reference electrode were a Pt rod and saturated calomel electrode (SCE), respectively. The working area of the electrode was fixed at 1 cm^2, and the distance between the working electrode and the light source (300 W xenon arc lamp coupled to an AM 1.5 G filter) was 15 cm. The surface photocurrent spectra (SPC) were collected by measuring the photocurrent under chopped light irradiation (20 s light/dark cycles).

Preparation of GO

GO was synthesized from natural graphite (~325 mesh, 99.95%) by a modified Hummers method: (1) The mixture of graphite powders (1.0 g), $K_2S_2O_4$ (0.5 g) and P_2O_5 (0.5 g) were put into an 80 °C solution of concentrated H_2SO_4 (98%, 10 ml) for 4 h. Then, the dark mixture was filtrated by deionized water for several times and dried in a stove; (2) The pre-oxidized graphite was put into 15 ml H_2SO_4 (98%), and 4 g $KMnO_4$ was gradually added with stirring and cooling with an ice-water bath; (3) 30 ml deionized water was added to the solution after stirring for 2 h at 35 °C; (4) The solution was kept at 85 °C for 30 min, and then, H_2O_2 (30%) was added to the solution until the color of the mixture turned to bright yellow. The GO was obtained after filtration, pickling, washing, and drying.

Morphology observation and characterization of $CuSbS_2$ quantum dots

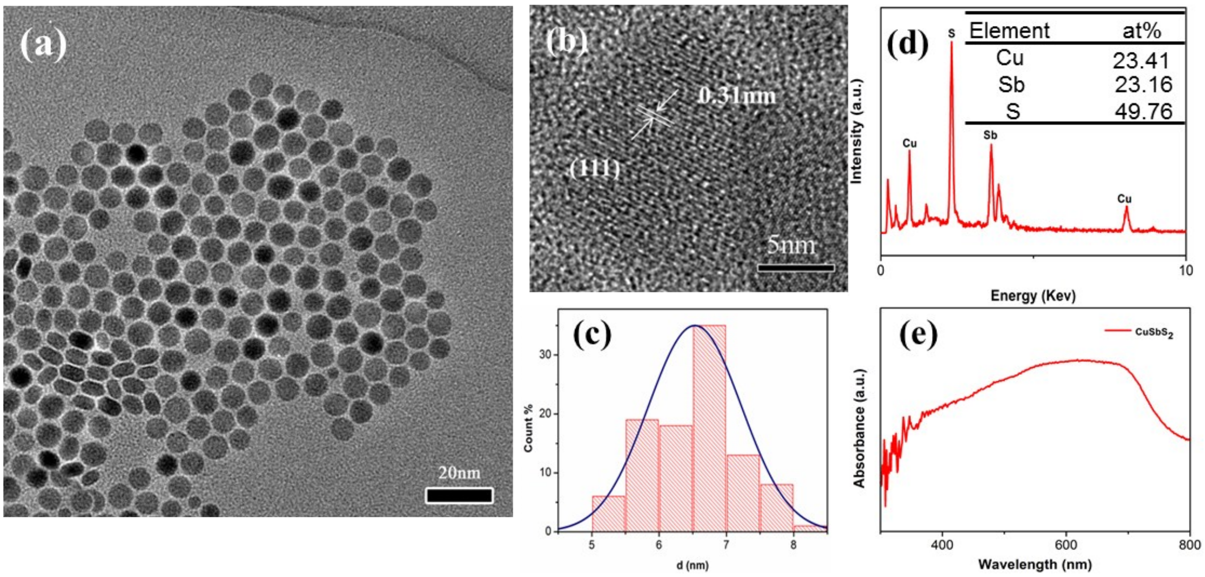

Fig. S1 $CuSbS_2$ quantum dots: (a) TEM image (low resolution), (b) TEM image (high resolution); (c) Size distribution histograms; (d) EDS profile; (e) UV-Vis spectra.

Fig. S1(a) shows the TEM images of the $CuSbS_2$ quantum dots, apparently, they are uniformly distributed without any agglomeration. HRTEM observation reveal that the interplanar distance of 0.31 nm is corresponding to (111) plane of the $CuSbS_2$ quantum dots (JCPDS 65-2416), as shown in Fig. S2(b). The average particle size of the $CuSbS_2$ quantum dots were calculated to be 6.7 nm (Fig. S2(c)), and its uniform particle size were correspond with the TEM image. The composition of $CuSbS_2$ quantum dots was characterized as well. The Cu/Sb/S molar ratio of 23.41: 23.16: 49.76 was obtained from the energy dispersive X-ray (EDX) spectroscopy (Fig. S2(d)). It can be seen that the light-absorption range of $CuSbS_2$ quantum dots covered from 300 to 800 nm, in addition, it shows extremely strong absorption in visible light region, this phenomena not only indicate its narrow band gap characteristic, but also reflect an extremely sensitive response to visible light, as shown in Fig. S2(e).

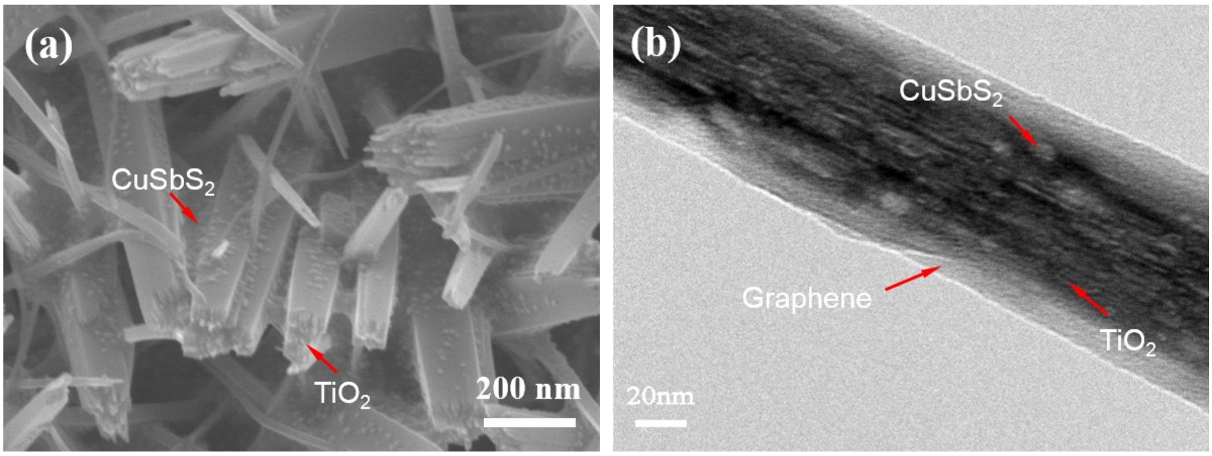

Fig. S2 (a) SEM images of 1D TiO_2 nanowires/0D $CuSbS_2$ quantum dots composite;

(b) TEM image of the 0D $CuSbS_2$ quantum dots/1D TiO_2 nanowires/2D graphene (mixed-dimensional) heterostructures.

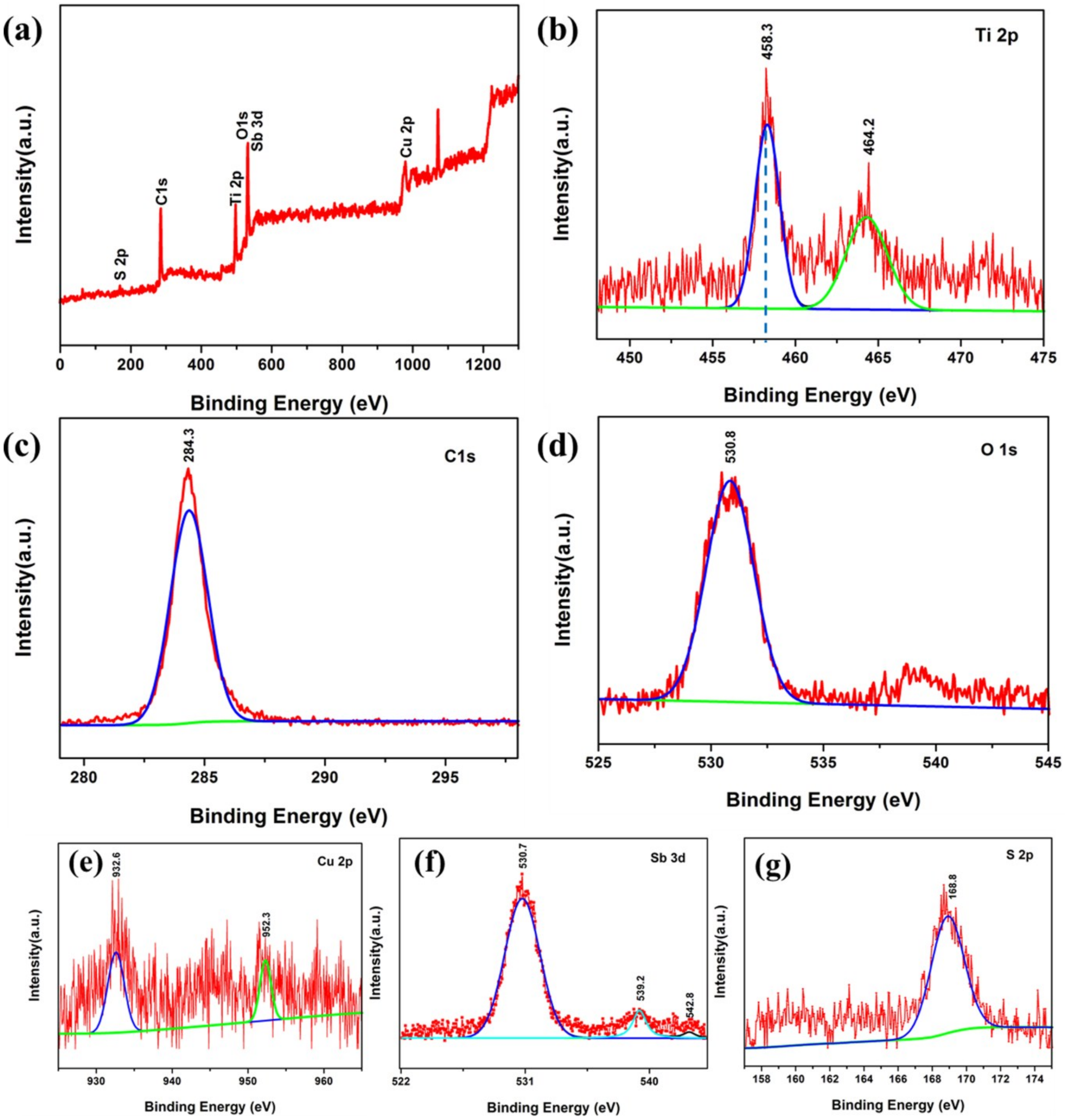

Fig. S3 XPS spectra of the 0D $CuSbS_2$ quantum dots/1D TiO_2 nanowires/2D graphene (mixed-dimensional) heterostructures: (a) Survey; (b) Ti 2p; (c) C 1s; (d) O 1s; (e) Cu 2p; (f) Sb 3d; (g) S 2p.

Fig. S3 illustrates the XPS profiles of the 0D $CuSbS_2$ quantum dots/1D TiO_2 nanowires/2D graphene (mixed-dimensional) heterostructures. Gaussian deconvolution of the high-resolution spectra was used to verify the chemical bond species and elemental chemical states. From the survey spectra, the sharp photoelectron peaks are obtained at the binding energies of 284.3 eV, 459.1 eV, 530.8 eV and 1021.9 eV, which indicate the existence of C 1s, Ti 2p, O 1s, Cu 2p, Sb 3d and S 2p elements, and the dominant intensity of C 1s is originated from graphene. Significantly, the characteristic peaks with binding energy of 459.1 eV (Ti

$2P_{3/2}$) and 464.6 eV (Ti 2p $_{1/2}$) are corresponding to the rutile phase of TiO_2 (Ti^{4+}),[1, 2] which exhibit a negative shift of 2 eV, and this phenomena suggest a strong interaction between graphene and the TiO_2 nanowires arrays, as show in Fig. S3(b). The high-resolution C 1s spectrum showed two main components, as shown in Fig. S3(c), i.e., the major peak at 284.3 eV is derived from the sp^2 carbon atoms of graphene, this result suggest that the thermal reduction of GO is efficient for removing the oxygen functional groups during the thermal treatment.[3] Significantly, the dominant peak intensity of C=C (284.3 eV) in graphene indicate the highly-ordered sp^2 carbon network, which is in good accordance with the results exhibit in the Raman spectra. As for the O 1s spectrum, the dominant peaks at 530.8 eV demonstrate a single chemical state of oxygen in the composite. The peak at 530.8 eV is attributed to the lattice oxygen in the TiO_2, which is corresponding to the C 1s species at 284.3 eV.[4] The featured peaks at 932.6 eV (Cu 2p $_{3/2}$) and 952.3 eV (Cu $2p_{1/2}$) are corresponding to the Cu^+ ($CuSbS_2$),[5] the peak at 530.7 eV (Sb $3d_{5/2}$) is consistent with chemical element state of Sb^{3+} ($CuSbS_2$),[6] and the characteristic peaks with binding energy of 168.8 eV (S $2p_{3/2}$) is corresponding to the S^{2-} ($CuSbS_2$).[7]

Table S1 A comparison study of the photoelectrochemical property of the 0D $CuSbS_2$ quantum dots/1D TiO_2 nanowires/2D graphene (mixed-dimensional) heterostructures photoanode in this work and previously reported TiO_2-based photonaodes

**Photoanode material**	**Photocurrent density**	**Light intensity**	**Testing condition**	**Reference**
TiO_2/rGO/NiFe-LDH	1.6 mA cm^{-2} (0.3 V vs. SCE)	100 mW cm^{-2}	0.5 M Na_2SO_4 (pH 6.8)	[5]
GO-decorated TiO_2	0.767 mA cm^{-2} (1.23 V vs. RHE)	100 mW cm^{-2}	0.5 M Na_2SO_4 (pH 7.0)	[7]
$BiVO_4$/Graphene/TiO_2	~0.13 mA cm^{-2} (0.8V vs. Ag/AgCl)	100 mW cm^{-2}	0.1 M K_2SO_4 (pH = 6.7)	[8]
AZO/TiO_2/Au	1.1 mA cm^{-2} at (0.6 V vs. SCE)	100 mW cm^{-2}	0.1 M Na_2SO_4 (pH = 6.8)	[9]
Disordered surface layer modified TiO_2	1.18 mA cm^{-2} (0.6 V vs. SCE)	100 mW cm^{-2}	0.5 M Na_2SO_4 (pH 6.8)	[10]
Carbon nitride quantum dots modified TiO_2	1.34 mA cm^{-2} (0.3Vvs.Ag/AgCl)	100 mW cm^{-2}	0.5 M Na_2SO_4 (pH 6.8)	[11]
α-Fe_2O_3 / TiO_2 nanorod array	3.39 mA cm^{-2} (1.23 V vs. RHE)	100 mW cm^{-2}	1 M NaOH aqueous solution	[12]
CdS quantum-dot /TiO_2-based	49.6 μA/cm^{-2} (0.4V vs. SCE)	100 mW cm^{-2}	3 M KOH aqueous solution	[13]
**TiO_2/graphene/$CuSbS_2$**	**3.91 mA cm^{-2} (0.3 V vs. SCE)**	**100 mW cm^{-2}**	**0.5 M Na_2SO_4 (pH 6.8)**	**This work**

References:

1. Y. Liu, H. Wang, K. Chen, T. Yang, S. Yang and W. Chen, *ACS Appl. Mater. Inter.,* 2019, **11**, 9573-9582.
2. N.T. Khoa, S.W. Kim, D. Yoo, S. Cho, E.J. Kim and S.H. Hahn, *ACS Appl. Mater. Inter.,* 2015, **7**, 3524-3531.
3. Y. Zhang, X. Li, X. Hua, N. Ma, D. Chen, H. Wang, *Scripta. Mater.,* 2009, **61**, 296-299.
4. L. Gao, W. Ren, F. Li and H. Cheng, *ACS nano,* 2008, **2**, 1625-1633.
5. F. Ning, M. Shao, S. Xu, Y. Fu,R. Zhang, M. Wei, D.G. Evans and X. Duan, *Energ. Environ. SCI.,* 2016, **9**, 2633-2643.
6. C. Zhai, M. Zhu, Y. Lu, F. Ren, C. Wang, Y. Du and P. Yang, *Phys. Chem. Chem. Phys.,* 2014, **16**, 14800-14807.
7. S.Y. Chae, P. Sudhagar, A. Fujishima, Y.J. Hwang and O. Joo, *Phys. Chem. Chem. Phys.,* 2015, **17**, 7714-7719.
8. S. Yousefzadeh, M. Faraji and A.Z. Moshfegh, *J. Electroanal. Chem.,* 2016, **763**, 1-9.
9. Y. Mi, L. Wen, R. Xu, Z. Wang, D. Cao, Y. Fang and Y. Lei, *Adv. Energy Mater.,* 2016, **6**, 1501496.
10. Z. Lian, P. Xu, W. Wang, D. Zhang, S. Xiao, X. Li, and G. Li, *ACS Appl. Mater. Inter.,* 2015, **7**, 4533-4540.
11. J. Su, L. Zhu, and G. Chen, *Applied Catalysis B: Environmental,* 2016, **186**, 127-135.
12. L. Jia, J. Xie, C. Guo and C. Li, *RSC Adv.*, 2015, **5**, 62611.
13. J. Du, M. Yang, F. Zhang, X. Cheng, H. Wu, H. Qin, Q. Jian, X. Lin, K. Li, D. Kang, *Ceramics International* 2018, **44**, 3099–3106
